# The Effect of Abrasive Waterjet Machining Parameters on the Condition of Al-Si Alloy

**DOI:** 10.3390/ma13143122

**Published:** 2020-07-13

**Authors:** Monika Kulisz, Ireneusz Zagórski, Jarosław Korpysa

**Affiliations:** 1 Department of Enterprise Organisation, Management Faculty, Lublin University of Technology, 20-618 Lublin, Poland; 2 Department of Production Engineering, Mechanical Engineering Faculty, Lublin University of Technology, 20-618 Lublin, Poland; i.zagorski@pollub.pl (I.Z.); j.korpysa@pollub.pl (J.K.)

**Keywords:** abrasive waterjet technology, Al-Si alloys, surface roughness, irregularities, specimen chamfering, microhardness, artificial neural networks, ANOVA statistical analysis

## Abstract

This paper analyses the effect of the abrasive waterjet cutting parameters’ modification on the condition of the workpiece surface layer. The post-machined surface of casting aluminium alloys, AlSi10Mg and AlSi21CuNi, was characterised in terms of surface roughness and irregularities, chamfering, and microhardness in order to reveal the effect that variable jet feed rate, abrasive flow rate, and sample height (thickness of the cut material) have on the quality of surface finish. From the analysis of the results, it emerges that the surface roughness remains largely unaffected by changes in the sample height h or the abrasive flow rate m_a_, whereas it is highly susceptible to the increase in the jet feed rate v_f_. It has been shown that, in principle, the machining does not produce the strengthening effect, that is, an increase in microhardness. Owing to the irregularities that are typically found on the workpieces cut with higher jet feed rates v_f_, additional surface finish operations may prove necessary. In addition, chamfering was found to occur throughout the entire range of speeds v_f_. The statistical significance of individual variables on the 2D surface roughness parameters, Ra/Rz/RSm, was determined using factorial analysis of variance (ANOVA). The results were verified by means of artificial neural network (ANN) modelling (radial basis function and multi-layered perceptron), which was employed to predict the surface roughness parameters under consideration. The obtained correlation coefficients show that ANNs exhibit satisfying predictive capacity, and are thus a suitable tool for the prediction of surface roughness parameters in abrasive waterjet (AWJ) technology.

## 1. Introduction—State of the Art

Machine components and semi-finished products are manufactured using a range of methods and types of machining operations. The 21st century manufacturing industries employ modern and cutting-edge machining techniques, including precision casting, plastic forming (die forging, cross-wedge rolling) [1], or high-speed milling (roughing, finishing, precision cutting) [2,3,4]. Increasingly often, the preparation of pre-products for subsequent processing involves abrasive water jet machining (AWJ)—one of the most dynamically developing technologies of modern manufacturing. Several terms for the technology have been proposed—abrasive waterjet machining (AWJM), abrasive waterjet cutting (AWJC), or abrasive waterjet technology (AWJT). AWJ machining is one of the most promising, non-conventional methods for shaping various types of structural materials, which is in a number of ways distinctly superior to other cutting methods [5]. Its advantages include high versatility with respect to the material being cut—it copes with virtually any metal/non-metal substrate except for diamond. AWJ machining of light alloys can be carried out at approximately twice the speed of steel. In addition, in the case of aluminium alloys, AWJ cuts through a range of thicknesses, from thin sheets (under 3 mm) to thick plates in the excess of 150 mm. The cut surfaces are smooth and show good quality of finish, rarely requiring further finishing treatment. High-pressure abrasive waterjet technology is undergoing constant evolution, which enables increasing its efficiency as well as the area and scope of applications [6].

The superiority of abrasive waterjet technology in comparison with conventional machining techniques is further bolstered by its other strengths [5,7]. The technology is capable of machining materials of any given hardness and can effortlessly shape intricate geometries. With respect to soft alloys, Mg or Al, the features of AWJ that are by far the most important markers of its capacity for handling these substrates [5] are as follows: no thermal effects, negligible cutting forces (reduced heat generation and no need for special technological equipment), and no dangerous dust or fumes during machining.

The process parameter that regulates the shape of the waterjet during cutting is water pressure; the waterjet stream and aerodynamic force rise with the increase in the pressure. This reduces the core zone of the waterjet and increases the jet exit angle. The jet geometry affects the depth of cut; when conical, an increase in the standoff distance will reduce the depth of cut. In addition, jet deflection occurs when a certain workpiece thickness is exceeded. The machining quality is particularly susceptible to the depth of cut; as the jet impact depth rises, the quality of the cut deteriorates. This may be explained by an uneven distribution of the kinetic energy of the abrasive medium (the energy is in the most part spent on removing the top layer of the workpiece, and the remaining energy is insufficient to provide a clear cut). As a result, machining tends to leave visible marks, striations, on a lower surface of cut [8].

The processing effectiveness is described and measured by various indicators, including efficiency; the quality of workpiece surfaces; technological parameters; or, alternatively, as a ratio of the amount of labour to the effect of that work [3]. In the aviation industry, the factors that are critical for production efficiency are, inter alia, a proper design of a component, machine-tool-workpiece-system rigidity, high-durability tooling, and optimal technological machining parameters that ensure high efficiency and adequate stability.

The chief focus of the efficiency-oriented investigations is to establish the effect that certain processing conditions have on the material removal rate and the geometry of the finished product. With respect to surface quality, researchers typically consider the impact of feed speed and standoff distance [9,10].

Abrasive waterjet technology is most widely studied in aluminium alloy machining. The scope of Al alloys tested in scientific investigations is quite extensive and includes a variety of classes, from nearly pure aluminium alloys (1xxx series) [11,12], to Al-Mg alloys (5xxx series) [11,13,14], Al-Mg-Si alloys (6xxx series) [11,15,16,17,18,19,20,21,22], and Al-Zn-Mg alloys (7xxx series) [11,23,24]. The selected referenced research is summarised in Table 1.

Computer simulation and mathematical modelling techniques are becoming widely employed in studies of machining processes and related phenomena, including the condition of the surface layer. The tendency applies to abrasive waterjet machining of soft alloys, as exemplified by Kolohan and Khajavi’s study [10,26] that utilised statistical regression in the analysis of 6063-T6 aluminium to study how various process parameters (water pressure, jet traverse rate, abrasive flow rate, and focusing nozzle diameter) affect the depth of cut. The model was verified using the analysis of variance (ANOVA) technique. Other studies [27] made use of the response surface methodology based on grey theory (g-RSM) by simultaneous optimisation aimed to predict optimal cutting data.

Kale et al. [28] investigated the material removal rate and surface roughness with the Taguchi and grey-relational analysis and combined it with analysis of variance (ANOVA) to determine the effect changing parameters on the analysed factors. The same method, Taguchi (ANOVA), was used by Maneiah et al., who examined metal matrix composites (MMCs) of Al-6061 [29]. Fuzzy logic and regression equations have also been employed to predict surface roughness in a number of studies, including the analysis of the abrasive waterjet machining of AZ91 magnesium alloy involving response surface methodology (RSM) [30].

With respect to surface roughness, in several works, it has been investigated with the implementation of neural networks, for instance, in the study of variable AWJ parameters [31]. The work in question attempted to establish the correlation between cutting parameters and surface roughness, which necessitated using the artificial neural network (ANN) and multiple regression tools based on Taguchi’s orthogonal array. In a similar study, by Zain et al. [32], regression modelling, SA optimisation, GA optimisation, integrated SA–GA-type1 optimisation, and integrated SA–GA-type2 optimisation served to predict the surface roughness parameter Ra in abrasive waterjet machining of Al 7075 alloy. In another instance, Zagórski et al. [5] analysed the condition of the AZ91D alloy workpiece surface by forecasting Ra, Rz, and RSm after cutting with the abrasive jet stream that was carried out for a variable set of parameters, specifically, the jet feed velocity v_f_ and the abrasive flow rate m_a_.

ANN-supported analyses of the effects of AWJ machining are not limited to Mg or Al alloys and include other metals, for example, steel, as evidenced by the study of Selvan et al. [33] or Ganowska et al. [34]. The studies referenced in the preceding paragraphs constitute only a small extract of an extensive body of scientific literature showing the use of ANN modelling. Other applications of the method include modelling surface roughness in milling [2,35], turning [36,37], or drilling [38].

For practical reasons, the research direction that should necessarily be explored is the study of the effect of input parameters of AWJ on the output model parameters: quality and accuracy [6]. What emerges from the study of literature is the notable paucity of published research material on the application of AWJ in the machining of Al-Si alloys.

Furthermore, it was found that an aluminium alloy with a low content of abrasive particles, such as the Si–AlMgSi0.5, has only once been an object of a scientific study [22]. The paper, however, did not address the post-machining condition of the Al-Si surface, as it focused on two indicators: width of the kerf and taper angle. The key observations were that the amount of energy is a decisive factor for the size and type of the cone-shaped cavity in the cut zone. It was thus resolved that the subject matter required deeper investigation, particularly given that the cut material in question has not been tested within the scope specified in our work. A further motivation is the practical implication of the undertaken study. Si-rich alloys are the source of machining problems rooted in tool wear or cutting edge degradation, contributing to a substantial rise in machining costs. These issues are effectively avoided when AWJ machining is performed, which additionally indicates that abrasive waterjet technology exhibits the necessary capacity to replace conventional machining methods in the preparation of Al or other soft pre-products for subsequent machining processes. This work provides evidence indicating that AWJ technology could decrease the cost-intensity of the process (tool/abrasive material costs), while positively impacting its efficiency.

## 2. Materials and Methods

**Figure 1:**
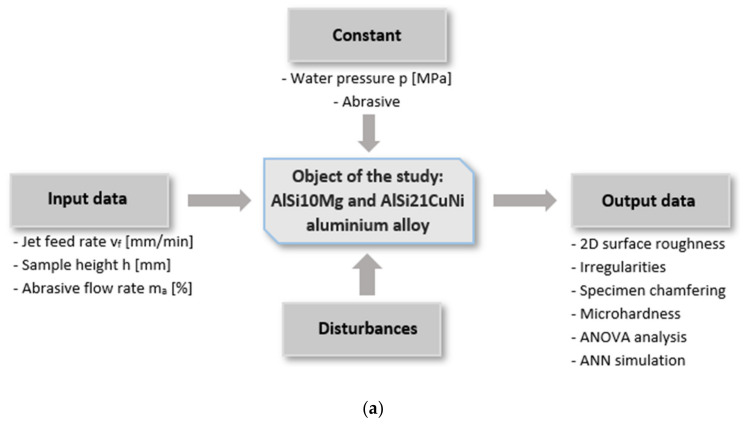

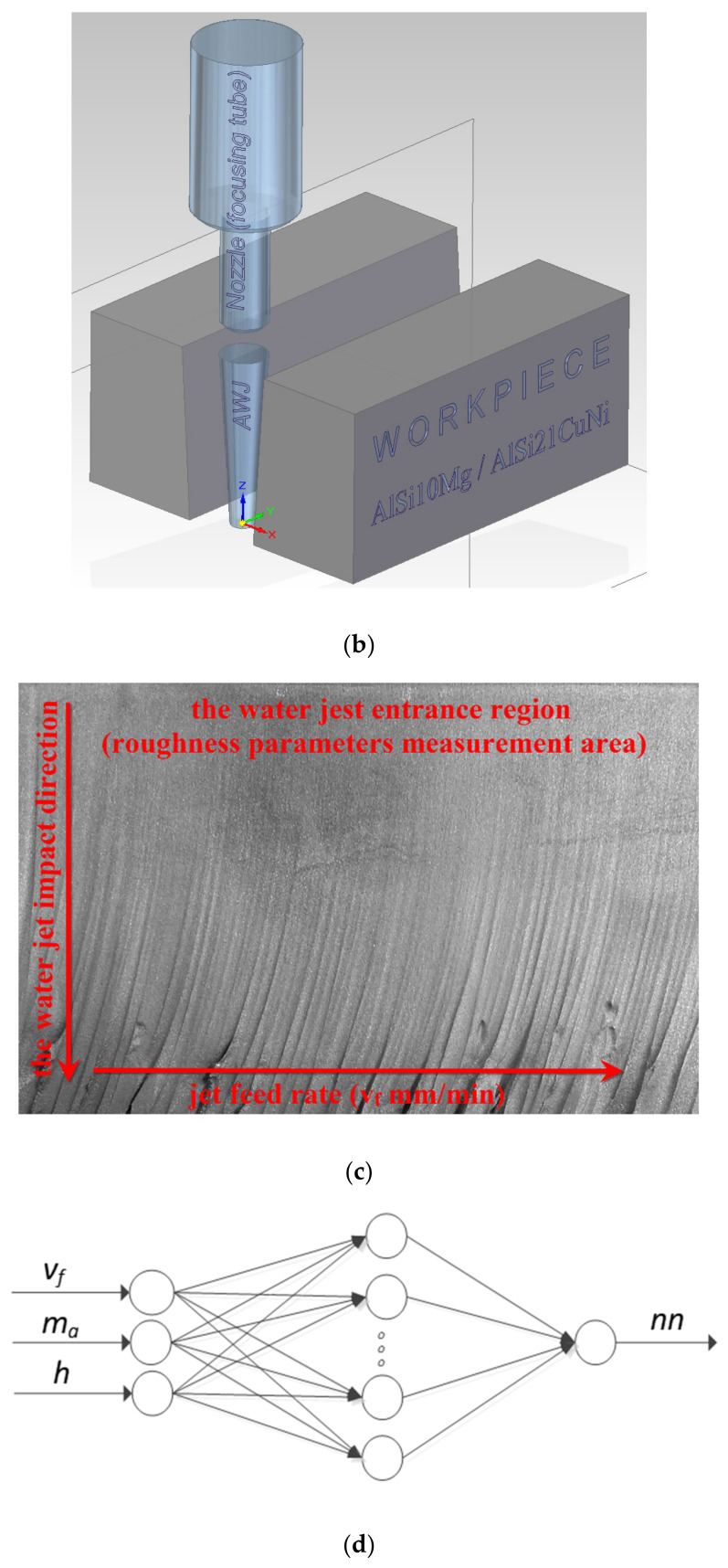
Schematics of (**a**) test flowchart, (**b**) abrasive waterjet (AWJ) visualisation, (**c**) measurement roughness parameters area, and (**d**) neural network for surface roughness parameter prediction. ANOVA, analysis of variance; ANN, artificial neural network.

The essential part of this work is the study of the abrasive waterjet machining of Si-enriched Al alloys AlSi10Mg and AlSi21CuNi with respect to process quality and efficiency. Figure 1 presents the test flowchart, the AWJ cutting visualisation, and the neural network predictive model.

In the course of the investigations, the testing procedures and methodology were carried out with strict adherence to an International Standard, PN-EN ISO 9013. The variable machining parameters were as follows: the abrasive flow rate m_a_, the jet feed rate v_f_, and the sample (pre-product) height h. The Al-Si alloy cutting process was performed with OPAL WATERJET COMBO (Legnica, Poland) cutting machine for plasma and waterjet cutting. The specimens were produced in the following sizes: AlSi10Mg 60 mm × 46/23/12 mm × 270 mm and AlSi21CuMn 60 mm × 46/23/12 mm × 154 mm.

The abrasive medium was a widely used blasting abrasive GARNET 80 mesh, which is natural sand characterised by low dustiness, low wear, and high hardness, whose sharp-edged particles provide good machinability properties [5]. Table 2 shows the constant AWJ cutting settings.

Table 3 shows the variable cutting parameters tested during AWJ machining. As in the case of the constant parameters, these were pre-determined from previous studies and the literature.

The machine-cut specimens were subjected to 2D roughness analysis. The surface of the cut was measured in five repetitions in two regions of the workpiece; at the waterjet entrance and in the middle of sample height (corresponding to the half of specimen thickness). The data obtained from the roughness measurements, performed using Hommel’s T1000 (Jena, Germany) roughness tester, have eventually enabled us to determine the optimal AWJ cutting settings, considering the surface quality. The 2D surface roughness measurement sampling parameters were as follows: ISO 16610-21:2013-02 filter (M1), sampling length, lr = 0.8 mm; evaluation length, ln = 4 mm; traversing length, lt = 4.8 mm; traverse feed velocity, and v_t_ = 0.5 mm/s.

Surface irregularities, that is, gaps between extreme peaks and valleys that were carved in the workpiece by the waterjet impacting its surface, were viewed using the optical system composed of the VHX 5000 digital microscope from Keyence (Osaka, Japan) equipped with VH-2 100R optics. The tilting angle on the post-machined surface was measured along the jet axis using the Vista ZEISS (Jena, Germany) coordinate measuring machine with auxiliary equipment: Renishaw (Wotton-under-Edge, UK) PH10M probe head with TP20 touch-trigger probe kit and Renishaw A-5003-0047 stylus (ball diameter d = 5 mm; effective working length l = 30 mm; ball weight of 2.57 g). The specimen tilting angle (chamfering) data were processed with the application of Power Inspect 4.3.5.1. The touch-and-learn mode enabled some automation of measurements; once performed manually, the measuring cycle was saved and repeated automatically on other specimens. The specimens were fixed on the measuring table using purpose-made clamps.

Further detail in the description of the post-machined surfaces was provided by microhardness testing. The procedure was performed using the Leco LM700AT (St. Joseph, MI, USA) microhardness tester with diamond pyramid-shaped indenter of a 136° included angle with a load of 5 g (0.05 HV) (Vickers method). The microhardness of the specimen was measured on the face and lateral surfaces; in the latter case, it was along the jet axis direction. The testing was fully compliant with international standards, PN-EN ISO 6507-1:2018-05.

In the predictive part of the study, the nonlinear technological process of abrasive waterjet machining was modelled to find Ra, Rz, and RSm parameters for two substrates: AlSi21CuNi and AlSi10Mg alloys. Given that the ANN had to account for three input parameters (cutting data) and two substrates, six different artificial network models were obtained. The network schematics are shown in Figure 1d, where “nn” in the output stands for a respective Ra, Rz, or RSm surface roughness parameter for a given alloy. To obtain the simplest network structure, the models contain a single hidden layer, three neurons in the input (the jet feed rate v_f_, the abrasive flow rate m_a_, and the sample height h), and one neuron in the output (Ra, Rz, and RSm).

It was resolved that the best-fitting networks to model the investigated relationships were the radial basis function (RBF) and multi-layered perceptron (MLP). The entire process was modelled in Statistica environment. The MLP was trained using three approaches: the BFGS (Broyden–Fletcher–Goldfarb–Shanno) method, the conjugate gradient, and the steepest descent training algorithm, whereas the RBFT algorithm was used for RBF. The activation functions were linear, exponential, logistic, tanh, and sinusoidal for the MLP network, and of the Gaussian distribution for RBF neurons in the hidden layer and a linear function for the output. Other testing parameters that were established experimentally were the number of hidden neurons (2 ÷ 15) and training epochs (150–300). The networks were modelled with 24 sets of machining parameters. The training dataset used 75% of the measurement results and the remaining 25% enabled validation. Testing sets were omitted owing to a small number of all datasets [2]. The suitability of particular networks was assessed using the following: training quality, validation quality, and training error determined by the method of least squares.

## 3. Results

### 3.1. Surface Roughness after AWJ Method

**Figure 2:**
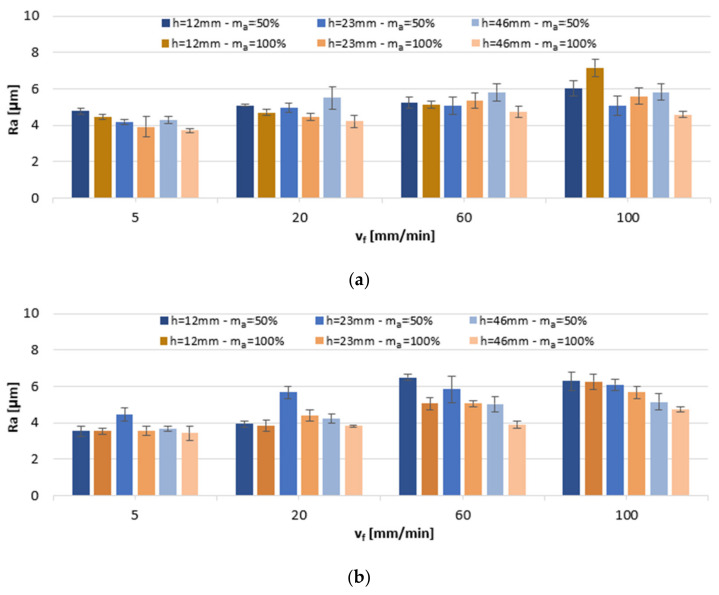
The impact of jet feed rate v_f_, sample height h, and abrasive flow rate m_a_ on Ra of post-machined alloys: (**a**) AlSi10Mg and (**b**) AlSi21CuNi.

The condition of the surface was first assessed from the perspective of the effect that variables v_f_, h, and m_a_ have on selected parameters of surface roughness. Figure 2 shows average Ra results measured on the surfaces of the AlSi10Mg and AlSi21CuNi alloys.

With respect to the AlSi10Mg alloy, the Ra parameter values were in the range of 3.71–7.16 µm. At a reduced abrasive flow rate (m_a_ = 50%), the smallest values were obtained for a medium sample height, h = 23 mm. At a full rate of abrasive flow (m_a_ = 100%), the lowest values were reported at the sample height h = 46 mm and the highest for the smallest height, h = 12 mm. Considering the second of the tested alloys, AlSi21CuNi, the Ra values were in the range of 3.44–6.49 µm. For AWJ cutting executed at a lower abrasive flow setting, the lowest values were recorded in samples h = 12 mm, in the range of v_f_ = 5–20 mm/min, whereas at a deeper cut, h = 46 mm, in the range of v_f_ = 60–100 mm/min. At full abrasive flow rate, m_a_ = 100%, the lowest values were obtained in the thickest specimens. For both alloys, average values increased with the rise in the cutting speed v_f_ and were lower in most cases at a full abrasive flow rate m_a_ = 100%. In comparison with the results for the AZ91D alloy [5], where the Ra parameter was in the range of 3–6 µm, it can be assumed that the obtained surface roughness characteristics are fully comparable, even though, in certain machining settings, the Ra in Si-enriched alloys approached 7 µm (Figure 2a—v_f_ = 100 mm/min, Figure 2b—v_f_ = 60–100 mm/min). Furthermore, our data converge with the results from the tests conducted with a resembling set of parameters (i.e., 5–7 µm at cutting speed 100 mm/min) on other soft alloys Al 2017, Al 5083, Al 6060, and Al 7075, investigated in previous studies [11].

**Figure 3:**
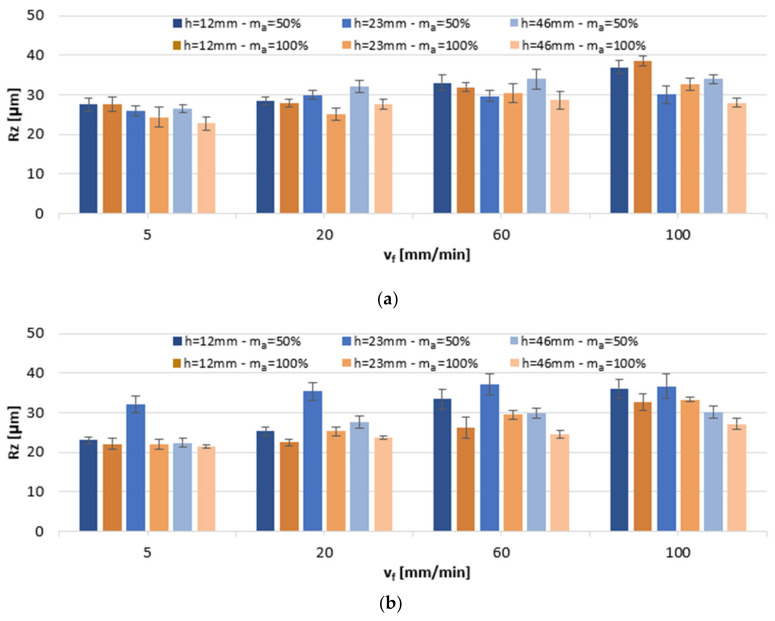
The impact of jet feed rate v_f_, sample height h, and abrasive flow rate m_a_ on Rz of post-machined alloys: (**a**) AlSi10Mg and (**b**) AlSi21CuNi.

The results in Figure 3 display the effect of variable cutting parameters on Rz. In AlSi10Mg alloym the values of Rz ranged between 22.79 µm and 38.60 µm. The smallest Rz readings at a 50% abrasive flow were recorded mostly for sample height h = 23 mm, while for higher abrasive flow, m_a_ = 100%, at a sample height h = 46 mm. Considering AlSi21CuNi alloys, the average Rz values ranged from 21.47 to 37.12 µm and, regardless of the abrasive medium flow, the lowest values were obtained predominantly in h = 46 mm specimens. It was found that, similarly to the Ra parameter, increasing the cutting speed v_f_ would produce a rougher surface (Rz) and that it was the full abrasive flow, m_a_ = 100%, that produced the smoother surface finish. We detected much higher values for Rz than those reported in previous works [5]. In the referenced study, Rz virtually never exceeded 35 µm in AZ91D alloy, while in our data for Al-Si alloys, Rz reached the level of up to 40 µm.

**Figure 4:**
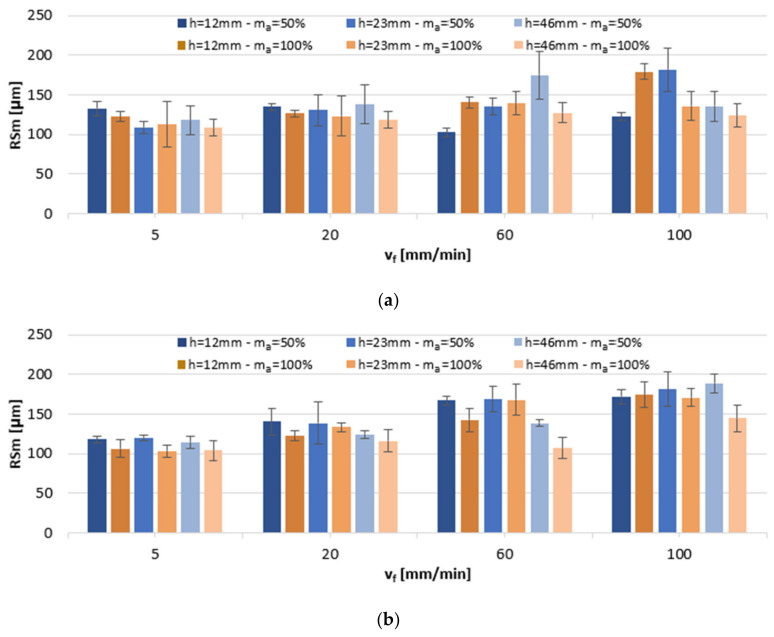
The impact of jet feed rate v_f_, sample height h, and abrasive flow rate m_a_ on RSm of post-machined alloys: (**a**) AlSi10Mg and (**b**) AlSi21CuNi.

Figure 4 presents the impact of variable machining factors on average RSm values. The surface roughness of AlSi10Mg alloy specimens varied between 109.2 and 181.8 µm, depending on the specific cutting data settings. At a reduced abrasive flow m_a_ = 50%, the lowest RSm values were recorded in h = 23 mm samples cut in the range of v_f_ = 5–20 mm/min, and at lower heights, h = 12 mm, in the range of v_f_ = 60–100 mm/min. The values of RSm were the lowest at m_a_ = 100% and sample height h = 46 mm. With respect to the other investigated workpiece material, AlSi21CuNi alloy, RSm ranged between 103.2 and 189.0 µm and attained the lowest values in h = 46 mm samples. The increase in the cutting speed was found to result in an increase in average RSm values on the surfaces of both alloys, whereas it was a higher abrasive flow rate that produced surfaces generally exhibiting lower RSm values. Values in a similar range (100–200 µm) were reported in the study of AZ91D [5].

**Figure 5:**
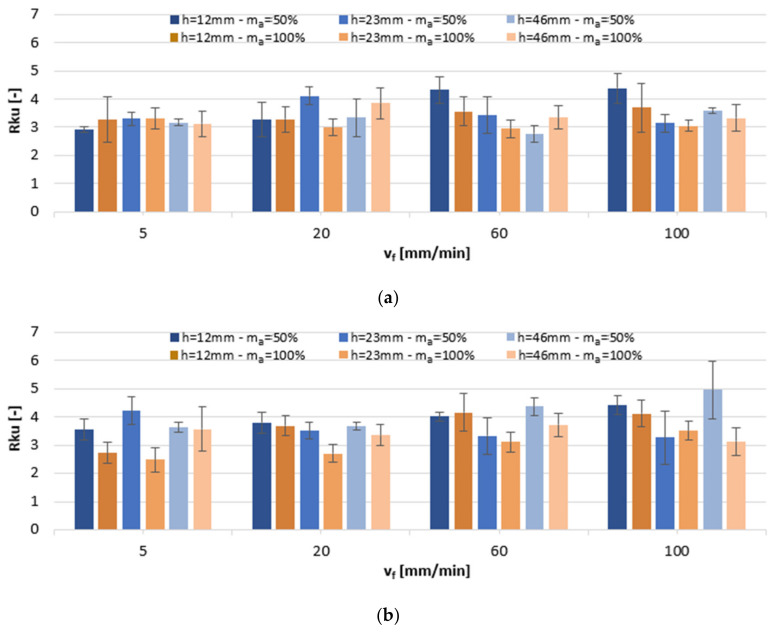
The impact of jet feed rate v_f_, sample height h, and abrasive flow rate m_a_ on Rku of post-machined alloys: (**a**) AlSi10Mg and (**b**) AlSi21CuNi.

Rku levels in response to changing AWJ cutting parameters are shown in Figure 5. On the AlSi10Mg alloy surfaces, the kurtosis values were close to 3, indicating normal peak distribution, which, in the case of mating surfaces, reduces the friction coefficient. This tendency was not displayed by h = 12 mm samples, whose Rku values were notably higher at a 50% abrasive flow rate. The values of Rku were found to exceed 3 on all AlSi21CuNi alloy specimens that were machined with abrasive flow rate m_a_ = 50%, which is indicative of sharp-peak surfaces. With respect to the full abrasive flow rate, m_a_ = 100%, Rku > 3, which could result from the increased cutting speed v_f_, further leading to the sharpening of peaks and valleys of the profile.

**Figure 6:**
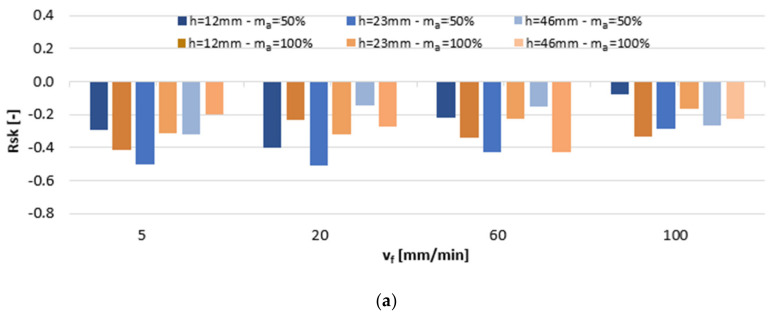

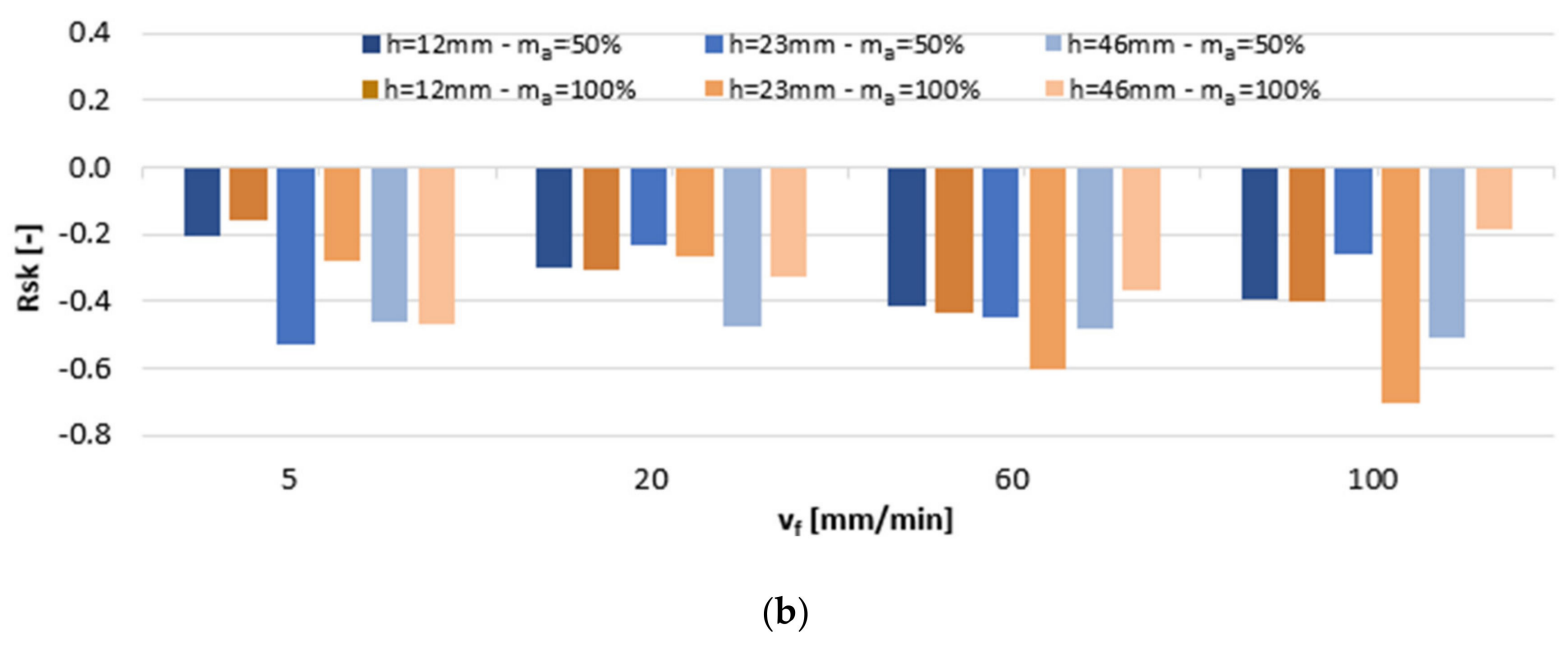
The impact of jet feed rate v_f_, sample height h, and abrasive flow rate m_a_ on Rsk of post-machined alloys: (**a**) AlSi10Mg and (**b**) AlSi21CuNi.

Figure 6 shows the impact of variable machining settings on the average values of the Rsk parameter. The surface of both alloys exhibited a flat plateaued finish of peaks, as indicated by Rsk < 0, which designates a lower coefficient of surface friction. Increasing the cutting speed v_f_ was in the majority of cases shown to increase kurtosis on the AlSi10Mg alloy surfaces, while on the AlSi21CuNi alloy surfaces, it caused their reduction.

### 3.2. Irregularities after AWJ—Microscopic Examination of Machined Specimen Surface

The accuracy of the AWJ machining was verified by the analysis of irregularities at the jet exit from the cut at the bottom of the workpiece. What is understood by “irregularities” is the distance between the highest peak and the lowest valley within a defined area at the bottom of the specimen. The irregularities were measured according to the standards set out in the respective norms, referenced in the preceding sections [5].

Table 4 shows surface irregularities expressed as numerical values. The results were obtained from the microscopic examination report for various sample heights—h: 46 mm, 23 mm, and 12 mm.

Table 4 displays an increase in the irregularities correlated with both higher jet feed rate (v_f_) and sample height (h). Furthermore, in AlSi10Mg alloys, the irregularity value is contained within the range of ≤0.2 mm for cutting speeds not exceeding v_f_ = 60 mm/min (except for h = 23 mm and m_a_ = 50%). These results imply that workpieces subjected to machining within the specified range of v_f_ may not require further post-treatment, which in turn leads to substantial time savings and cost reduction already at the pre-production stage.

In general, higher irregularity errors were recorded on surfaces of AlSi23CuNi alloys, which is most likely associated with an increased quantity of abrasive grain (mainly Si and Ni), which may impede the decoherence of the material in the area of cut (in addition, higher hardness and tensile strength).

The rate of irregularity is by far most substantial at sample height h = 46 mm, the maximum jet feed rate v_f_ = 100 mm/min, and abrasive flow rate m_a_ = 50%. The respective maximum irregularities for AlSi10Mg and AlSi21CuNi alloys are 0.944 mm and 1.893 mm. Comparing the results from Table 4 with former data [5], it can be seen that, for the AZ91D alloy, an acceptable machined surface quality (surface irregularities < 0.2 mm) is achieved given that the cutting speed does not exceed v_f_ = 40 mm/min (m_a_ = 50%) or v_f_ = 80 mm/min (m_a_ = 100%). In our tests, the satisfactory condition of Al-Si alloy surfaces in comparable-height specimens was produced under the condition that the cutting speeds did not exceed v_f_ = 60 mm/min. Therefore, the observed difference is quite significant.

### 3.3. Specimen Surface Chamfering after AWJ

**Figure 7:**
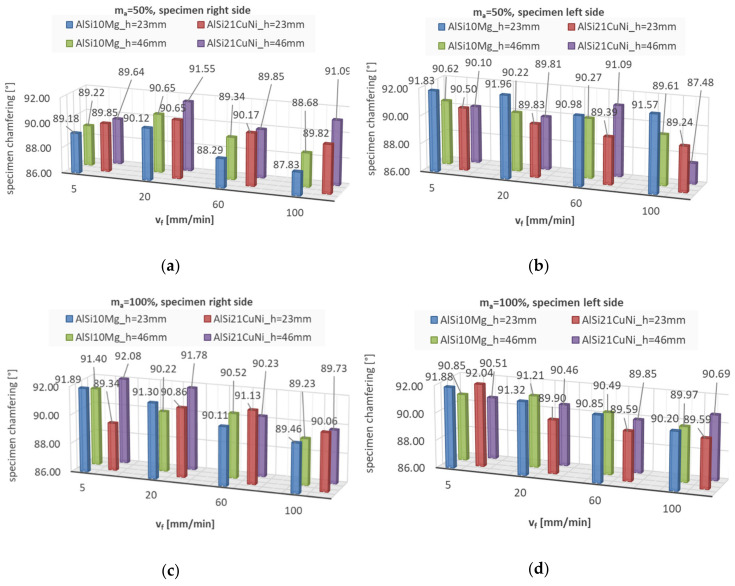
Specimen chamfering by abrasive waterjet machining depending on the side of impact and abrasive flow rate (m_a_ = 50% and 100%): (**a**) right side specimen m_a_ = 50%, (**b**) left side specimen m_a_ = 50%, (**c**) right side specimen m_a_ = 100%, (**d**) left side specimen m_a_ = 100%.

While cutting, abrasive waterjet interacts with two surfaces, the left and right side of the specimen. Each cut produces two surfaces that have been machined at the same feed rate. Figure 7 presents specimen chamfering on two sides impacted at by the abrasive waterjet (left/right). The measurement methodology is consistent with previously performed tests presented before [5].

Chamfering is represented by the data in the graphs above. The specimen surface angle on the left of the cut is typically greater than 90°. However, the inclination angle of the specimen surface on the right of the cut is less than 90°. Although, in essence, slight changes in the chamfering angle were found to be technologically insignificant, the issue could be subjected to further statistical analysis with a view of verification. Considering the least favourable scenarios, the absolute values of specimen chamfer were as follows: AlSi10Mg m_a_ = 50%—2.17°, AlSi10Mg m_a_ = 100%—1.88° and AlSi21CuNi m_a_ = 50%—2.52°, and AlSi21CuNi m_a_ = 100%—2.08°. For planeness and parallelism of the specimen sides, a certain finishing treatment would be advised. The Al-Si data concur well with previous findings [5], where the absolute tilt angle was 1.94° for the abrasive flow rate of 50% and 1.09° for the flow rate of 100%. A slight discrepancy can be seen in AZ91D alloy, which, at m_a_ = 100%, displayed generally lower chamfering angle values.

### 3.4. Specimen Surface Microhardness

**Figure 8:**
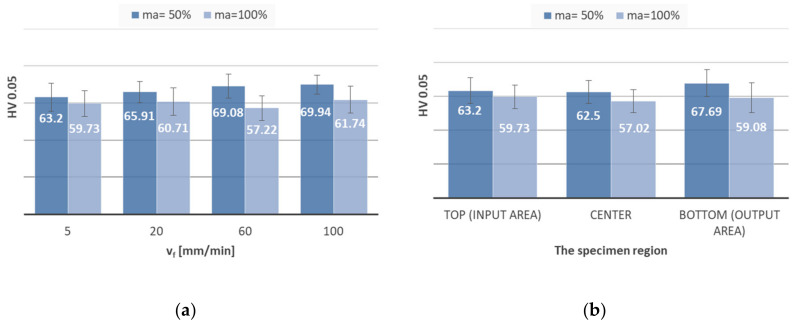
Microhardness: (**a**) with variable jet feed rate v_f_ and (**b**) in different specimen region v_f_ = 5 mm/min.

The microhardness readings were analysed only in AlSi10Mg alloy workpieces owing to inadequate cut quality in the AlSi21CuNi alloy cut material. Figure 8a displays the changes in surface microhardness following the modification of the jet feed rate. Additionally (Figure 8b), the microhardness analysis was carried out in three different regions of specimens machined at uniform speed (v_f_ = 5 mm/min), where the measurements were possible (no striation or irregularities).

Jet feed rate (Figure 8a) was found not to cause a significant increase in average microhardness. According to Figure 8b, the microhardness levels were to a large extent constant across the three regions of the workpiece. A possible explanation for these observations is that the cut surface of Al-Si alloy workpieces was not hardened as a result of the abrasive grain impact. In comparison [5], the AZ91D magnesium casting alloy developed higher values of surface microhardness (approximately 100 HV). At 70 HV, the AlSi10Mg alloy cut material investigated here was hence softer.

### 3.5. Statistical Analysis

The statistical significance of the data, and thus the importance of individual variables on the surface roughness parameters, was assessed using a factorial ANOVA (significance level α = 0.05). Prior to the analysis, the normal distribution of data was confirmed using the Shapiro–Wilk test. Although it emerged from the Levene test that the homogeneity of variance hypothesis was not true for all analysed groups, in homoscedastic groups, ANOVA exhibited good robustness to the heterogeneity of variance [39].

ANOVA results for the AlSi10Mg alloy are given in Table 5. Variables were shown to be significant (*p* < 0.05) for all the surface roughness parameters, except for RSm, in which case the sample height and the abrasive flow rate proved not significant. In addition, certain factors were found to be interdependent although, considered separately, they were statistically insignificant.

ANOVA results for the AlSi21CuNi alloy (Table 6) show the significance of all variable factors (*p* < 0.05) on the analysed surface roughness parameters. There are also interactions between the factors being changed that indicate the simultaneous influence of several factors on the surface roughness. The interaction does not occur only in relation to the RSm parameter between cutting speed and abrasive output, although these variables are statistically significant individually.

Table 6 presents the ANOVA results for the values of Ra, Rz, and RSm roughness parameters obtained on the surfaces of the AlSi21CuNi alloy.

The cutting speed v_f_, sample height h, and abrasive flow rate m_a_ have a statistically proven influence on the values of the Ra, Rz, and RSm parameters of AlSi10Mg and AlSi21CuNi aluminium alloys.

### 3.6. Numerical Modelling of Surface Roughness Parameters with Artificial Neural Networks after AWJ Method

The data from the measurements carried out on the AlSi21CuNi and AlSi10Mg aluminium alloy specimens have allowed us to describe the surface of the specimens after they were machined using AWJ technology. The parameters that were used for the purpose, Ra—arithmetical mean roughness of the profile, Rz—maximum height of profile, and RSm—mean width of profile elements, were subsequently used as input for the simulation of surface roughness parameters using MLP and RBF artificial neural networks.

The suitability of networks for modelling relationships between sets of data is typically assessed from the analysis of the network quality indicators: learning quality, validation quality, learning error, and validation error derived from the least-squares method. A total of 200 networks were generated to handle each modelled scenario. The quality of the networks was evaluated with the indicators above to select a better-suited model, MLP or RBF. Table 7 presents the network quality parameters for AlSi21CuNi and AlSi10Mg alloys with regards to Ra, Rz, and RSm. Training and validation quality and errors, as well as the hidden and output layer activation functions, are given. From the data on the AlSi21CuNi alloy, it is shown that the best-fitting network considering Ra was RBF 3-8-1 with eight neurons (network no. 1); for Rz, it was MLP 3-2-1 with two neurons (network no. 2); while for the RSm parameter, it was RBF 3-14-1 with fourteen neurons (network no. 3). In the case of the AlSi10Mg alloy, the networks were as follows: Ra—network no. 4, RBF 3-15-1 (network with fifteen neurons); Rz—network no. 5, MLP 3-14-1 (network with fourteen neurons); RSm—network no. 6, RBF 3-12-1 (network with twelve neurons). On aggregate, RBF networks provided a closer fit of results for Ra and RSm, while the MLP model performed better in the prediction of Rz.

**Figure 9:**
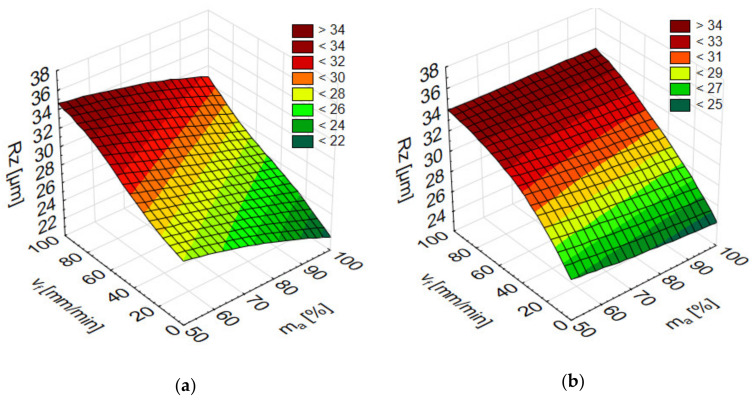

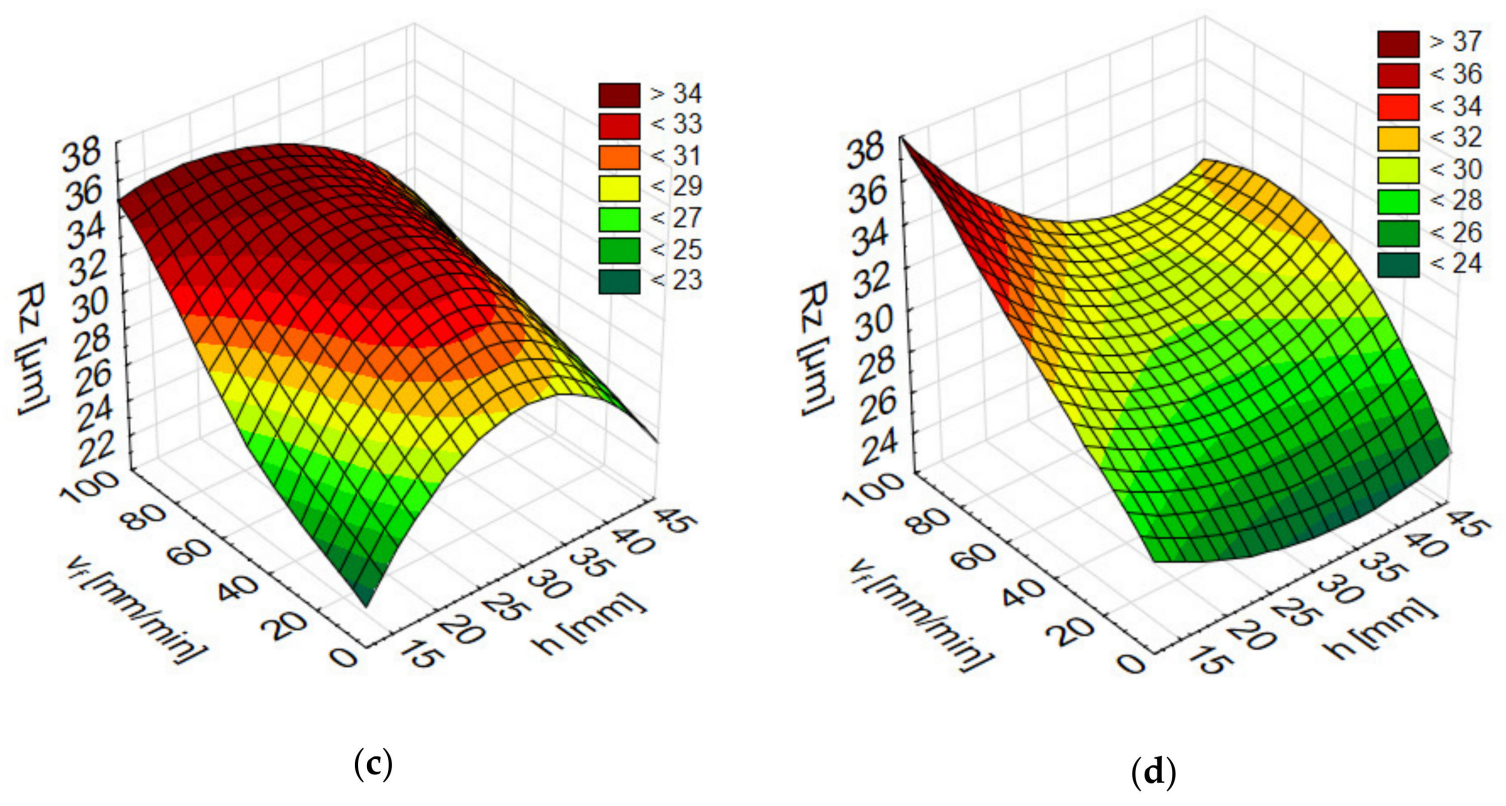
Numerical results of surface roughness parameters Rz after AWJ depending on the jet feed rate v_f_ and abrasive flow rate m_a_ for (**a**) AlSi21CuNi alloy (MLP 3-2-1) and (**b**) AlSi10Mg alloy (MLP 3-14-1), and depending on the jet feed rate v_f_ and sample height h for (**c**) AlSi21CuNi alloy (MLP 3-2-1) and (**d**) AlSi10Mg alloy (MLP 3-14-1).

**Figure 10:**
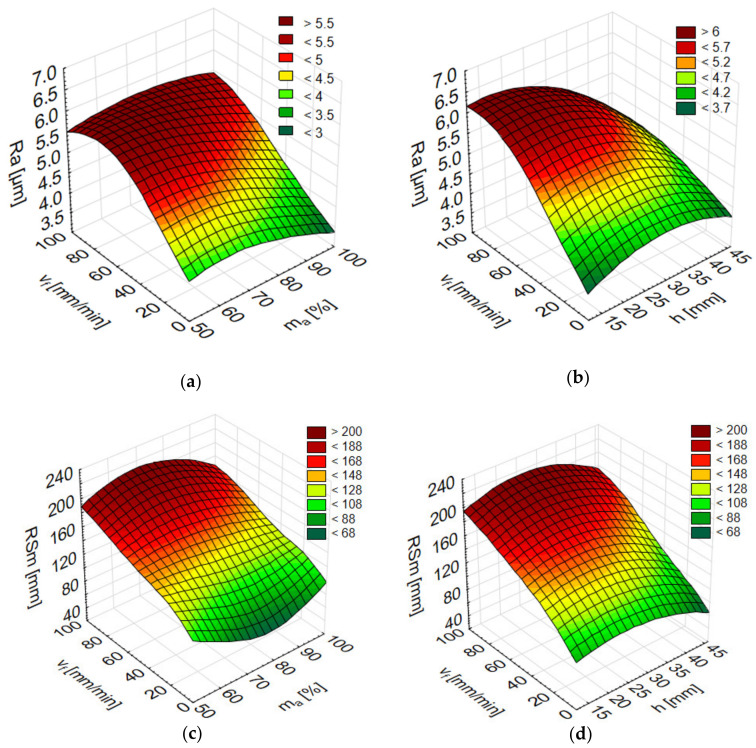
Numerical results after AWJ for AlSi21CuNi alloy for surface roughness parameters Ra (RBF 3-8-1) depending on the jet feed rate v_f_ and abrasive flow rate m_a_ (**a**), as well as depending on the jet feed rate v_f_ and sample height h (**b**), and surface roughness parameters RSm (RBF 3-14-1) depending on the jet feed rate v_f_ and abrasive flow rate m_a_ (**c**), as well as depending on the jet feed rate v_f_ and sample height h (**d**).

The 3D graphs below show the numerical results from the simulation of surface roughness parameters for the Al alloys following machining with the abrasive waterjet technology. Given that three input parameters were considered, they are presented in pairs in two figures: the jet feed rate v_f_ and abrasive flow rate m_a_, and the jet feed rate v_f_ and sample height h. Figure 9 shows the surface roughness results for Rz for AlSi21CuNi (MLP 3-2-1) and AlSi10Mg (MLP 3-14-1). Figure 10 concerns the Ra (RBF 3-8-1) and RSm (RBF 3-14-1) parameters simulated for AlSi21CuNi alloy. The experimentally established quantities, v_f_, m_a_, and h, were entered in Statistica as inputs and provided the basis for simulating the output parameters.

**Figure 11:**
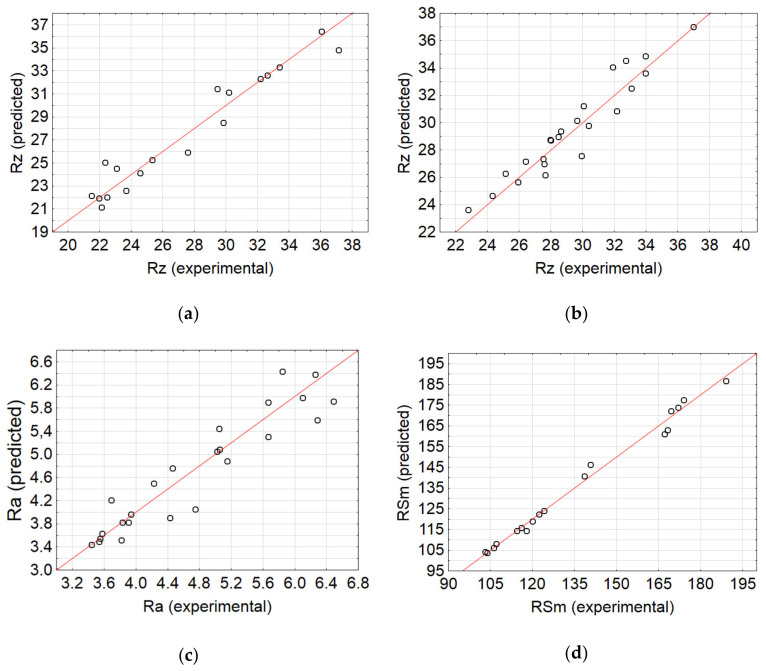
Comparison of artificial neural network (ANN) results with experimental measurements of surface roughness parameters Rz (**a**) AlSi21CuNi alloy (MLP 3-2-1) and (**b**) AlSi10Mg alloy (MLP 3-14-1); (**c**) surface roughness parameters Ra AlSi21CuNi alloy (RBF 3-8-1); and (**d**) surface roughness parameters RSm AlSi21CuNi alloy (RBF 3-14-1).

Table 8 presents the correlation coefficients of Ra, Rz, and RSm in AlSi21CuNi and AlSi10Mg alloys. The results confirm that the artificial neural networks are good predictors of surface roughness parameters. In addition, the correlation between the experimental and simulation data for networks visualised in Figure 9 and Figure 10 is further shown in Figure 11.

The conclusion that arises from the graphs and figures above is that the networks exhibit good predictive capacity, which is additionally underlined by the coefficient of correlation R^2^ > 0.9, and thus are effective tools for simulating surface parameters after abrasive waterjet machining processes. The data produced by the artificial networks could provide the foundation for subsequent processing and incorporation into numerical models of machining processes.

## 4. Conclusions

The experimental and mathematical findings from the study are significant in several major respects.

1. In the case of both aluminium alloys, the increase in jet feed rate v_f_ led to the deterioration of the surface smoothness (higher roughness parameters), and the sample height h modification was not found to produce a constant effect on the investigated parameters.
2. Although there was no strong correlation between the abrasive flow rate and the surface roughness characteristics of the specimens, there was a slight tendency towards lower roughness levels on the part of workpieces machined with full abrasive flow rate m_a_ = 100%.
3. AlSi23CuNi alloy exhibited higher susceptibility to machining errors, confirmed by higher surface irregularity rates. The AlSi10Mg alloy provides better machinability and, under specific conditions (v_f_ ≤ 60 mm/min), the pre-products machined using AWJ technology may not require additional finishing machining.
4. Jet feed rate v_f_ has a low impact on the mean microhardness of AlSi10Mg alloy specimens cut with the reduced abrasive flow rate m_a_ = 50%, and is virtually negligible in the case of full abrasive flow rate m_a_ = 100%. Constant microhardness levels were displayed at different measurement points on the specimens (v_f_ = 5 mm/min).
5. In the analysed range of sample heights h, workpieces showed a certain degree of chamfering that could require post-treatment aimed to reduce the undesirable chamfer angle.
6. From the statistical analysis, it emerges that, in the majority of cutting scenarios, the change in the jet feed rate v_f_, the sample height h, and the abrasive flow rate m_a_ will have a strong effect on the levels of Ra, Rz, and RSm on the surfaces of the AlSi10Mg and AlSi21CuNi alloys. In addition, particular technological parameters directly interact with each other.
7. Artificial neural network modelling may be an effective tool for predicting surface roughness parameters. The correlation coefficient R^2^ for both alloys is R^2^ > 0.93; therefore, the trained networks are sound predictors of the surface roughness characteristics with respect to the tested materials.
8. ANN models determine relationships between input machining parameters (v_f_, m_a_, h) and output (Ra, Rz, RSm) surface roughness parameters of AlSi21CuNi and AlSi10Mg alloys. This enables computing the AWJ machining data that will ensure the optimal cutting performance and results even in the absence of preliminary test runs.

## Figures and Tables

**Table 1:** Summary of abrasive waterjet cutting light alloys.

Materials	Machining Conditions	Research Object	Reference
Al 1050	Traverse speed v_t_ = 100–600 mm/min	Surface topography Surface roughness Machinability	[11]
Al 2017			
Al 5083			
Al 6060			
Al 7075			
Al 1060	Velocity v = 0.6–1.5 mm/s	Surface roughness	[12]
	Pressure P = 25–33 MPa		
	Stand-off distance h = 0.5–2 mm		
	Abrasive diameter d = 80–200 #		
Al 5083-H32	Abrasive mesh size 80–120 # Jet impingement angle 70–90°	Surface morphology	[14]
		Surface topography	
		XRD peak	
		Residual stress	
		Micro hardness	
Al 6060	Cutting speed V = 200–1000 m/min	Surface topography Surface roughness Surface roughness prediction	[15]
	Material thickness S = 6–20 mm		
	Abrasive flow Q = 300–400 g/min		
	Measurements position 1–3		
Al 6061-T6	Stand-off distance h = 2–10 mm Mask thickness t_m_ = 1–2 mm Mask opening W_m_ = 50–150 µm Number of passes n = 1–120 Nozzle angle Ɵ = 45–90°	Channel cross-sectional shape Instantaneous normalized centreline erosion rate Surface roughness Surface waviness	[17]
Al 6061 T651	Feed rate v_f_ = 50–100 mm/min Thickness g = 1–10 mm	Width of processed surface	[18]
		Inclination angle	
		Deviation from perpendicularity	
		Surface roughness	
Al 6061 T651	Cutting method	Width of processed surface Deviation from perpendicularity Inclination angle Surface roughness	[20]
Al 6063-T6	Traverse rate V = 30–90 mm/min Abrasive flow rate M = 0.5–4.5 g/s Water pressure P = 100–250 MPa Focusing tube size F = 0.76–1.6 mm Orifice size O = 0.25–0.35 mm	Depth of cut Kerf width Surface roughness	[21]
Al 7075	Traverse speed v_t_ = 30–150 mm/min Water pressure P = 100–300 MPa Stand-off distance SoD = 1–3 mm	Surface roughness	[23]
Al 7475	Feedrate v = 3000–5000 mm/min	Surface texture	[24]
	Pressure P = 40–50 kpsi	Fatigue life	
	Standoff distance s = 10–25 mm	Stress	
	Passess 1–4	Material removal rate	
Al-alloy (lack of material grade)	Material thickness t = 15–30 mm Traverse speed v = 37–350 mm/min Abrasive mass flow rate m_a_ = 100–390 g/min	Surface roughness	[25]
AZ91D	Jet feed velocity v_f_ = 5–180 mm/min Abrasive material flow rate m_a_ = 50–100%	Irregularities Surface chamfering Surface roughness Microhardness	[5]

**Table 2:** Constant technological parameters of abrasive waterjet (AWJ) cutting.

**Nozzle (focusing tube) diameter—d_o_**	0.7 (mm)
**Abrasive (size)**	Garnet 80 mesh
**Standoff distance**	3 (mm)
**Nozzle width**	60 (mm)
**Water pressure—p**	350 (MPa)

**Table 3:** Variable technological parameters of AWJ cutting.

Workpiece	Sample Height h (mm)	Abrasive Flow Rate m_a_ (g/min)	Jet Feed Rate v_f_ (mm/min)
AlSi10Mg	46	500 (g/min) i.e., –100% 250 (g/min) i.e., –50%	5–100
	23		
	12		
AlSi21CuNi	46		
	23		
	12		

**Table 4:** Quantified surface irregularities after AWJ machining.

Al-Si Alloys	v_f_ (mm/min)	h (mm)					
		m_a_ = 50%			m_a_ = 100%		
		46	23	12	46	23	12
**AlSi10Mg**	5	0.125	0.235	0.063	0.095	0.118	0.117
**AlSi21CuNi**	5	0.172	0.123	0.226	0.13	0.414	0.33
**AlSi10Mg**	20	0.166	0.273	0.068	0.117	0.189	0.221
**AlSi21CuNi**	20	0.173	0.153	0.241	0.134	0.519	0.474
**AlSi10Mg**	60	0.259	0.353	0.084	0.227	0.23	0.07
**AlSi21CuNi**	60	0.924	0.159	0.537	0.255	0.19	0.587
**AlSi10Mg**	100	0.944	0.352	0.199	0.577	0.202	0.111
**AlSi21CuNi**	100	1.893	0.334	0.79	0.944	0.237	0.62

**Table 5:** Analysis of variance (ANOVA) for different roughness parameters—Ra, Rz, and RSm (AlSi10Mg alloy).

Effect		DF	Ra				Rz				RSm			
			SS	MS	F	p	SS	MS	F	p	SS	MS	F	p
**v_f_**	**(A)**	3	35.41	11.80	92.370	0.000	986.40	328.80	118.230	0.000	13,587.00	4529.00	16.885	0.000
**h**	**(B)**	2	6.42	3.21	25.100	0.000	197.40	98.70	35.490	0.000	199.00	100.00	0.371	0.691
**m_a_**	**(C)**	1	3.11	3.11	24.340	0.000	105.10	105.10	37.800	0.000	701.00	701.00	2.613	0.109
**AB**		6	7.99	1.33	10.420	0.000	204.30	34.00	12.240	0.000	10,444.00	1741.00	6.489	0.000
**AC**		3	2.88	0.96	7.520	0.000	27.20	9.10	3.260	0.025	582.00	194.00	0.724	0.540
**BC**		2	7.93	3.97	31.040	0.000	137.10	68.60	24.650	0.000	9113.00	4556.00	16.987	0.000
**ABC**		6	3.49	0.58	4.550	0.000	68.50	11.40	4.110	0.001	14,311.00	2385.00	8.892	0.000
**Error**		96	12.27	0.13			267.00	2.80			25,750.00	268.00		
**Total**		119	79.51	25.09			1993.00	658.50			74,687.00	14,474.00		

**Table 6:** ANOVA for different roughness parameters—Ra, Rz, and RSm (AlSi21CuNi alloy).

Effect		DF	Ra				Rz				RSm			
			SS	MS	F	p	SS	MS	F	p	SS	MS	F	p
**v_f_**	**(A)**	3	72.13	24.04	218.720	0.000	1346.85	448.95	158.230	0.000	61,880.00	20,627.00	116.640	0.000
**h**	**(B)**	2	15.46	7.73	70.310	0.000	649.49	324.75	114.450	0.000	7172.00	3586.00	20.280	0.000
**m_a_**	**(C)**	1	10.45	10.45	95.060	0.000	721.33	721.33	254.220	0.000	6810.00	6810.00	38.510	0.000
**AB**		6	12.58	2.10	19.070	0.000	161.46	26.91	9.480	0.000	5760.00	960.00	5.430	0.000
**AC**		3	3.13	1.04	9.490	0.000	53.64	17.88	6.300	0.001	367.00	122.00	0.690	0.559
**BC**		2	1.02	0.51	4.620	0.012	128.87	64.44	22.710	0.000	1249.00	624.00	3.530	0.033
**ABC**		6	2.53	0.42	3.830	0.002	100.51	16.75	5.900	0.000	3236.00	539.00	3.050	0.009
**Error**		96	10.55	0.11			272.39	2.84			16,976.00	177.00		
**Total**		119	127.83	46.40			3434.54	1623.85			103,450.00	33,445.00		

**Table 7:** Multi-layered perceptron (MLP) and radial basis function (RBF) networks for surface roughness parameters Ra, Rz, and RSm in AlSi21CuNi and AlSi10Mg alloys.

Network No.	Network Name	Quality (Training, %)	Quality (Validation, %)	Error (Training)	Error (Validation)	Activation (Hidden)	Activation (Output)
**AlSi21CuNi alloy**							
Arithmetical mean roughness of the profile, Ra							
1	RBF 3-8-1	95.24	90.28	0.049	0.093	Gaussian	Linear
Maximum height of the profile, Rz							
2	MLP 3-2-1	96.99	96.49	0.755	0.984	Logistic	Linear
Mean width of profile elements, RSm							
3	RBF 3-14-1	99.48	94.09	4.088	53.73	Gaussian	Linear
**AlSi10Mg alloy**							
Arithmetical mean roughness of the profile, Ra							
4	RBF 3-15-1	99.86	91.31	0.001	0.164	Gaussian	Linear
Maximum height of the profile, Rz							
5	MLP 3-14-1	98.54	89.61	0.247	1.409	Exponential	Sinus
Mean width of profile elements, RSm							
6	RBF 3-12-1	98.99	88.56	4.769	63.98	Gaussian	Linear

**Table 8:** Correlation R^2^ for surface roughness parameters Ra, Rz, and RSm for AlSi21CuNi and AlSi10Mg alloys.

Alloys Grade	AlSi21CuNi			AlSi10Mg		
Surface Roughness Parameters	Ra	Rz	RSm	Ra	Rz	RSm
Network	RBF 3-8-1	MLP 3-2-1	RBF 3-14-1	RBF 3-15-1	MLP 3-14-1	RBF 3-12-1
Correlation R^2^	0.9384	0.9694	0.9719	0.9433	0.9646	0.9564
